# Pattern destabilization and emotional processing in cognitive therapy for personality disorders

**DOI:** 10.3389/fpsyg.2015.00107

**Published:** 2015-02-23

**Authors:** Adele M. Hayes, Carly Yasinski

**Affiliations:** Department of Psychological and Brain Sciences, University of DelawareNewark, DE, USA

**Keywords:** personality disorders, dynamic systems, associative networks, cognitive therapy, psychotherapy research

## Abstract

Clinical trials of treatments for personality disorders can provide a medium for studying the process of therapeutic change with particularly entrenched and self-perpetuating systems and might reveal important principles of system transition. We examined the extent to which maladaptive personality patterns were destabilized in a trial of cognitive therapy personality disorders (CT-PD) and how destabilization was associated with emotional processing and treatment outcomes. Dynamic systems theory was used as a theoretical framework for studying change.

**Method:** Participants were 27 patients diagnosed with Avoidant or Obsessive Compulsive Personality Disorder (AVPD or OCPD), who completed an open trial of CT-PD. Raters coded treatment sessions using a coding system that operationalizes emotional processing, as well as cognitive, affective, behavioral, and somatic components of pathological (negative) and more adaptive (positive) patterns of functioning. Pattern destabilization (dispersion) scores during the early phase of treatment (phase 1: session 1–10) and the schema-focused phase (phase 2: session 11–34) were calculated using a program called GridWare.

**Results:** More pattern destabilization and emotional processing in the schema-focused phase of CT-PD predicted more improvement in personality disorder symptoms and positive pattern strength at the end of treatment, whereas these variables in phase 1 did not predict outcome.

**Conclusion:** In addition to illustrating a quantitative method for studying destabilization and change of patterns of psychopathology, we present findings that are consistent with recent updates of emotional processing theory and with principles from dynamic systems theory.

## INTRODUCTION

For researchers interested in the science of change, psychotherapy for entrenched patterns of psychopathology can provide a context for revealing some basic principles of human change. Effective psychotherapy can be viewed as a way to perturb self-perpetuating and disabling patterns to facilitate new learning and more adaptive functioning. Personality disorders, by definition, are longstanding maladaptive patterns with interacting cognitive, affective, behavioral, and somatic components that are highly interconnected and resistant to change (American Psychiatric Association [APA], 2013). Avoidant and obsessive–compulsive personality disorders (AVPD and OCPD) epitomize two emotion regulation strategies that are associated with a number of forms of psychopathology – avoidance and repetitive, unproductive analysis and processing, such as worry and rumination (Hayes et al., 1996; Watkins, 2008; Kashdan and Rottenberg, 2010). Cognitive therapy for personality disorders (CT-PD; Beck et al., 2004) and related schema-based therapies (e.g., Young et al., 2003; Arntz, 2012) are designed to reduce regulation strategies that inhibit change and to dislodge pathological patterns that maintain personality disorders. Thus, clinical trials of treatments for personality disorders can provide a medium for studying the therapeutic change process with particularly entrenched and self-perpetuating problems. We examined the extent to which maladaptive personality patterns were destabilized in a trial of CT-PD (Beck et al., 2004), and whether destabilization was associated with treatment outcomes and with emotional processing, a key hypothesized mechanism of therapeutic change. We apply some basic principles from dynamic systems theory as a theoretical framework for the study of change.

### BASIC PRINCIPLES OF DYNAMIC SYSTEMS THEORY RELEVANT TO PSYCHOTHERAPY

A dynamic systems perspective, which has been applied across sciences such as physics, biology, ecology, chemistry, and political science involves the study of relatively stable patterns, called attractors, as well as system destabilization and the process by which new attractors develop and stabilize (Thelen, 1995). The principles and general approach of dynamic systems theory can inform the study of how effective therapy moves individuals from disabling and rigid patterns to more flexible and adaptive ones (Hayes et al., 2007c; Schiepek and Perlitz, 2009; Salvatore and Tschacher, 2012). We illustrate a relatively simple approach for studying concepts from dynamic systems theory and apply it to the study of change in CT-PD (Beck et al., 2004). It is important to note that we distill some basic principles from the science of dynamic systems that can inform psychotherapy research (for more comprehensive presentations, see Lewis, 2005; van Geert and Steenbeek, 2005; Granic and Hollenstein, 2006; Salvatore and Tschacher, 2012), but these are theoretical constructs and not the same as a true application of dynamic systems analysis and modeling. Nonetheless, the framework and methods that we describe can place the study of therapeutic change in the context of a broader science of change. This approach can also apply to the investigation of other types of treatment and patterns of psychopathology.

A dynamic system consists of components that constantly interact with each other and with internal and external processes to form patterns that change and evolve over time (Thelen, 1995). An adaptive system maintains a dynamic tension between stability and variability. Stabilizing forces maintain the coherence or integrity of a system, whereas variability provides the flexibility necessary for adaptation, growth, and change (Hollenstein et al., 2013). When a dynamic system self-organizes, the components settle into preferred and relatively stable patterns, called *attractor states.* The system tends to return to these patterns when perturbed. Attractors that are activated repeatedly over time and contexts are particularly stable. When attractors are entrenched, a significant amount of energy and perturbation is required to move a system from these preferred states. Attractors that are less developed or have been destabilized are more sensitive to perturbation and thus are more easily changed.

Dynamically stable systems undergo constant perturbation related to internal dynamics and interactions with the environment. Stabilizing or inhibitory forces maintain system coherence and integrity by absorbing or assimilating perturbations, keeping the system organized around the same attractor state(s). When challenges are too great to assimilate, change is often not gradual and linear, but rather is characterized by disturbance and increased variability in system behavior, which can facilitate changes in system organization called *phase* or *order transitions* (Kelso et al., 1993; van Geert and van Dijk, 2002; Salvatore and Tschacher, 2012).

Perturbation studies in dynamic systems research have documented two early indicators of system transition: (1) a period of increased variability in system behavior called *critical instability* (van der Maas and Molenaar, 1992; Kelso, 1997; Vallacher et al., 2002; Schiepek et al., 2003; Schiepek and Strunk, 2010), and (2) a period of *critical slowing*, which is an increase in the time to recover from perturbation that reflects attractor stability and resilience (Scheffer et al., 2012). These indicators of impending transition are reliably quantified by the extent of variance in system behavior and temporal (lag-1) autocorrelation (extent to which the system becomes more and more like its past state; Dakos et al., 2012a,b). The study of system behavior in the vicinity of these early indicators can reveal: (1) the nature of the interactions among system elements, (2) system response to perturbation and challenge, (3) the emergence and break down of attractors, (4) the relative flexibility and rigidity of the system, and (5) the probability of change (Hollenstein et al., 2013). System dynamics can be understood on multiple, interacting levels and time scales, from moment-to-moment fluctuations in human behaviors to constellations of personality traits that occur over long periods of time across a variety of contexts (Hollenstein et al., 2013).

During periods of fluctuation, the system is destabilized and therefore more flexible and open to new information and exploration of potentially more adaptive configurations. System flexibility is conceptualized as curvilinear in that too much or too little flexibility is associated with worse functioning, whereas moderate levels are likely to reflect the balance of system integrity and openness to change (Lunkenheimer et al., 2011; Hollenstein et al., 2013). A system that is too rigid is characterized by patterns that perseverate and repeat over time and are insensitive to shifts in contextual demands, all of which inhibit adaptation.

A period of “flickering” (Dakos et al., 2013) or oscillating between alternative attractors (e.g., old and new patterns) can precede or accompany transition, until the system settles into a new dynamically stable state, marked by decreased variability in system behavior and increased temporal autocorrelation (Thelen and Smith, 1994; Kelso, 1997; van Geert and van Dijk, 2002; Scheffer et al., 2012). A new attractor can be strengthened and generalized by repeated activation across multiple contexts. If more adaptive, this new attractor can then inhibit or compete with the old attractor state(s) to prevent a return to less adaptive functioning.

### DYNAMIC SYSTEMS CONCEPTS AND COGNITIVE-BEHAVIORAL THERAPY

Although not framed in the language of dynamic systems theory, key theories of change in cognitive-behavioral therapies (CBT) refer to constructs that can be understood from this perspective. For example, pathological associative networks, such as fear networks (Lang, 1977; Foa and Kozak, 1986), depressive networks or interlocks (Teasdale, 1999; Dozois and Beck, 2008), and the cognitive-affective-behavioral nodes and patterns of personality disorders (Young and Lindemann, 2002; Beck et al., 2004) can be conceptualized as attractors that are central targets of change in CBT.

Successful therapy is thought to involve the activation of these pathological patterns, together with exposure to corrective information and new experiences that induce dissonance. Consistent with dynamic systems principles, this disturbance challenges patients to develop new cognitive-affective-behavioral-somatic patterns rather than assimilate new information into old patterns. Destabilization of pathological patterns can facilitate new learning, a shift in meaning and affective response, and an integration of cognitive and affective experiences, often called *emotional processing* (Greenberg, 2002; Foa et al., 2006). This therapeutic processing involves approaching previously avoided or difficult experiences without becoming immersed in rumination, worry, venting, and other repetitive and unproductive forms of processing (Watkins, 2008). Emotional processing (also called cognitive-emotional processing) has been proposed by researchers across theoretical orientations to be a common mechanism of change, with applications across a range of treatments and clinical disorders (Foa and Kozak, 1986; Greenberg, 2002; Whelton, 2004; Foa et al., 2006; Carey, 2011; McCarthy et al., 2013; Hayes et al., 2014). Emotional processing is likely to be apparent at points of destabilization and system transition, as we have found in the treatment of depression with an exposure-based cognitive therapy (Hayes et al., 2007a; Holtforth et al., 2012).

Recent developments in human and animal learning theory also highlight the importance of developing and strengthening new associative networks and patterns, which can function like new attractors and compete with pathological patterns to reduce the risk of relapse (Bouton, 2002; Foa et al., 2006; Craske et al., 2008; Schiller et al., 2008; Schiepek et al., 2015). Depression researchers similarly have begun to emphasize not only destabilizing depressive patterns, but also generating and consolidating new, more positive and adaptive patterns (Dunn, 2012; Carl et al., 2013). For instance, Dozois et al. (2009) highlight that patients treated with cognitive therapy (combined with pharmacotherapy) showed both a decrease in the interconnectivity of negative interpersonal schemata and an increase in interconnectivity of positive interpersonal schemata. In contrast, those who received pharmacotherapy alone did not show such changes in connectivity. These authors suggest that the development of what can be conceptualized as a new attractor might account in part for the prophylactic effects of cognitive therapy. In a small sample patients who received exposure therapy for obsessive-compulsive disorder, Schiepek et al. (2013) also demonstrated that new patterns and qualitative shifts in functioning occurred during periods of increased disturbance, and further that these new patterns were associated with therapeutic changes in patterns of neuronal activation. In short, therapeutic change is likely to involve disrupting old, well-worn patterns and developing new, more adaptive configurations of cognition, emotions, behaviors, and somatic functioning that, with repetition across contexts, evolve into new attractors.

### RIGIDITY, FLEXIBILITY, AND CHANGE IN PERSONALITY DISORDERS

A dynamic systems framework may be particularly relevant when conceptualizing personality disorders and their treatment. Modern theorists propose that personality is a complex dynamic system, rather than a static grouping of traits or tendencies (Cloninger et al., 1997; Cervone, 2004). Extending his earlier cognitive-affective personality systems theory (CAPS; Mischel and Shoda, 1995, 1998) to treatment, Mischel (2004) contends that personality is more than the associations between single situations and responses and is better understood as relatively stable and predictable patterns that emerge over time. The challenge of therapy from this perspective is to identify the situations that trigger the patterns, change the relationships among the elements and the “processing dynamics,” decrease automaticity, and increase openness to modification (p. 194). Cervone’s (2004) knowledge-and-appraisal personality architecture (KAPA) and Read et al.’s (2010) “neural network model” of personality also suggest that personality is best understood by the dynamic interaction among its internal elements (e.g., cognitive, affective, behavioral) and between these elements and the external environment. Borsboom and Cramer’s (2013) network approach similarly conceptualizes psychopathology as a causal system of functionally interrelated symptoms that have settled into a pathological equilibrium (see also Schmittmann et al., 2013).

Personality disorders are characterized by dysfunctional personality traits or dimensions that are relatively stable across time and situations (American Psychiatric Association [APA], 2013). These disorders require therapists to treat problems at the level of patterns or networks, given the pervasiveness of the problems and the high rates of comorbidity with other disorders that further solidify the patterns (Clark, 2009). Young and Lindemann (2002) propose that “early maladaptive schemas” (EMSs), or deeply entrenched patterns of cognition, affect, and behavior, underlie the rigidity of personality disorders. EMSs are thought to stem from adverse early life experiences and to be maintained by perceptual biases and maladaptive behavioral tendencies that feed back into and strengthen these schemas. Beck et al. (2004) emphasize the importance of fully activating the cognitive-affective-motivational programs that form maladaptive personality patterns, exploring their historical antecedents, and introducing corrective information to destabilize old patterns and facilitate cognitive restructuring and emotional processing. Thus, schema-focused treatments are multimodal in their focus and target broad, maladaptive patterns of functioning. Recent evidence suggests that schema-focused approaches are associated with significant improvement in personality disorders (Leichsenring and Leibing, 2003; Arntz, 2012). In addition, change in schemas and symptomatology can mutually reinforce each other and contribute to the development of more adaptive patterns of functioning (Lobbestael et al., 2007; van Vreeswijk et al., 2014).

The task of therapy for Cluster C (anxious, fearful) personality disorders is to destabilize the maladaptive patterns that maintain the disorders and increase flexibility, which has been proposed to be a fundamental aspect of mental health (Kashdan and Rottenberg, 2010). For instance, in a time series of an individual patient with avoidant personality disorder (AVPD) and comorbid depression, Maurer et al. (2011) illustrated how more instability of problematic patterns was associated with transition points in the course of therapy and better outcome. The treatment of personality disorders might involve inducing two types of variability: (1) *opening and loosening* pathological patterns early in treatment by providing a strong treatment rationale, case conceptualization, and a supportive treatment context, as well as building resources and instilling hope and motivation; and (2) *destabilizing* pathological patterns by exposing the person to corrective information and experiences and facilitating emotional processing. Both should be marked by an increase in the variability of patterns of cognitive, affective, behavioral, and somatic functioning, but the variability early in treatment might set the conditions for change, whereas the destabilization in the schema-focused phase might predict more substantial shifts in personality symptoms and facilitate the development of more adaptive patterns (Hayes et al., 2007a, 2014).

In previous research examining data from this trial of CT-PD (Beck et al., 2004), some forms of disruption and variability predicted later improvement in personality symptoms. Strauss et al. (2006) found that “rupture-repair” episodes (disruptions in the therapeutic relationship that can provide corrective information and facilitate change) were associated with more improvement in personality disorder and depressive symptomatology at the end of treatment. Variability in self-esteem within the first 10 sessions of treatment was also associated with better treatment outcomes (Cummings et al., 2012). These findings suggest that increased variability in intra- and interpersonal functioning may be an important marker of change in CT-PD. Although promising, these studies examined disruption of single variables (self-esteem, the therapeutic alliance) rather than pathological and more adaptive patterns of functioning, the focus of the current study.

### THE CURRENT STUDY

We examined change in cognitive-affective-behavioral-somatic patterns of patients with AVPD and OCPD, who received CT-PD (Beck et al., 2004). This therapy can be conceptualized as a perturbation in that it is designed to activate, challenge, and loosen multimodal patterns of personality functioning, which can be conceptualized as attractors. We describe a coding system that can be used to create pathological and more adaptive pattern variables with four components: cognitive, affective, behavioral, and somatic functioning. We illustrate how a freely available computer resource, GridWare (Lamey et al., 2004; Hollenstein, 2007), can be used to capture qualitatively and quantitatively the dynamics of pattern activation across the course of therapy.

We predicted that more destabilization of the pattern of pathological personality functioning, particularly in the schema-focused phase of CT-PD, would be associated with more symptom change and also with the emergence of a more positive, adaptive pattern at the end of treatment. More emotional processing during this period of destabilization was also expected to predict better outcomes. We explored whether the disturbance of old patterns and emotional processing were both important in the change process, or whether one or the other was primary.

## MATERIALS AND METHODS

### DATA SOURCE

Outcome data for this study were drawn from an archived open trial of CT-PD for AVPD and/or OCPD. The details of the trial have been described in an earlier publication (Strauss et al., 2006); we present below the design and outcome variables relevant to the present study. Audiotaped therapy sessions from the trial were coded to create the negative (personality disorder-related) and positive (more adaptive) patterns, as well as the emotional processing variable.

### PARTICIPANTS

Potential participants were administered the Structured Clinical Interview for the DSM-III-R (SCID; Spitzer et al., 1990a) and the Structured Clinical Interview for the DSM- III-R Personality Disorders (SCID-II; Spitzer et al., 1990b) at intake. As noted in the Strauss et al. (2006) description of the trial, a review of the original assessments revealed that all patients also met criteria for SCID-II for DSM-IV (First et al., 1997). Exclusion criteria were active suicidality, substance dependence within the past year, psychosis, bipolar disorder, schizotypal or borderline personality disorder, or organic dysfunction. Thirty patients in that trial met diagnostic criteria for a primary diagnosis of AVPD (*n* = 22) or OCPD (*n* = 8) and completed the session 34 symptom assessment. In addition, 75% met criteria for comorbid major depressive disorder, 56% for a comorbid anxiety disorder, and 28% of those with a primary diagnosis of AVPD or OCPD also met criteria for the other personality disorder. Patients were allowed up to 52 sessions that occurred across 12–16 months. On average, participants attended 29.74 sessions (SD = 18.85).

Twenty-seven of the 30 patients had symptom data at pretreatment and week 34 (which was used as the posttreatment score) and had audible session tapes during that period. The mean age of participants was 34 years old (SD = 9.30). The majority of patients were female (15 female, 12 male), single or divorced (63% single/divorced, 33% married), and 8% were ethnic minorities. All but one participant had some college education.

### PERSONALITY DISORDER SYMPTOMS

Personality disorder symptoms were assessed by the SCID-II (Spitzer et al., 1990b). Interviewers were postdoctoral psychologists with extensive training in structured interviewing and blind to patients’ diagnosis and progress in therapy. Interviewers probed and rated the presence of each personality disorder symptom on a 3-point scale (0 = *absent*, 1 = *subthreshold*, 2 = *present*). Unweighted kappa coefficients for inter-rater agreement for AVPD and OCPD diagnoses were 0.94 and 0.69, respectively, which fall in the good to excellent range of agreement (Landis and Koch, 1977). Personality disorder severity ratings were obtained by totaling the individual symptom scores for each disorder to yield dimensional scores that corresponded to patients’ primary diagnosis (AVPD or OCPD). The SCID-II was administered at intake, session 17, session 34, and at the last treatment session.

### THERAPISTS AND TREATMENT OUTCOME

Fourteen therapists (2 predoctoral, 12 doctoral-level), who were previously trained in cognitive therapy at the Center for Cognitive Therapy at the University of Pennsylvania, received additional training in CT-PD (Beck et al., 1990). CT-PD is similar to Beck’s cognitive therapy for depression (Beck et al., 1979) in its focus on dysfunctional schemata, cognitive-affective-behavioral connections, and teaching skills to modify schematic vulnerabilities. In addition, CT-PD places more emphasis on examining the historical roots of problems, interpersonal patterns, the therapeutic alliance, and eliciting in-session affect. The early phase of treatment focuses on symptom reduction (roughly the first 10 sessions), especially related to mood and anxiety disorders, and then the focus moves to schema level change (after session 10). We therefore examined process variables in the symptom reduction phase (phase 1) and in the schema-focused phase (phase 2: sessions 11–34).

Therapists received one hour of individual supervision for every two hours of therapy and attended weekly group supervision meetings and monthly case conferences. In addition, the Revised Cognitive Therapy Rating Scale (Blackburn et al., 2001) was used by raters blind to type of Cluster C diagnosis and treatment outcome. One session was sampled and rated from phase 1 and one session from phase 2 for each patient. The mean therapist competence ratings were above the established threshold for competence (for details see Strauss et al., 2006).

Outcome analyses for all patients who completed any personality and depression symptom assessments after initial intake were reported in Strauss et al. (2006). *T*-tests of pre to posttreatment differences revealed that CT-PD was associated with significant improvement in personality and depression symptoms with large effect sizes [SCID-II: mean difference = 6.59, SD = 3.31, 95% CI = 5.27–7.91, *t*(29) = 10.16, *p* < 0.001; Cohen’s *d* = 1.98; Beck Depression Inventory (BDI; Beck et al., 1961): mean difference = 8.60, SD = 8.45, 95% CI = 5.89–11.30; *t*(29) = 6.43, *p* < 0.001; effect size *d* = 1.02]. Only 6% met diagnostic criteria for AVPD or OCPD at posttreatment, and although 75% met criteria for a comorbid mood disorders at intake, only 37% met criteria at posttreatment.

### CODING OF CT-PD SESSIONS

Coders were three doctoral-level clinical psychology graduate students and one bachelor-level research assistant. All were blind to patient diagnosis, session number, and treatment outcome. Coders were trained to criterion with practice coding for approximately 10 h. After reaching criterion agreement (intraclass correlation coefficient; ICC = 0.80), two coders rated each session. Coders were paired with each other an equal number of times. Weekly to biweekly meetings were held to review discrepancies and prevent rater drift.

The *CHANGE coding system* (Hayes et al., 2006) was used to code the content of therapy sessions in this trial of CT-PD. The coding system includes a range of variables thought to be important in the therapeutic change process. The variables relevant to the current study assess cognitive, affective, behavioral, and somatic aspects of functioning, as well emotional processing. Each variable is coded on a scale from 0 to 3 (0 = *not present or very low*, 1 = *low*, 2 = *medium*, 3 = *high*). Variables are not mutually exclusive and can co-occur.

All sessions were audiotaped, and each session was coded independently by two of the four raters. Sessions were 50 min in duration. Because each patient had up to 52 session tapes and coding is labor-intensive, we coded every other session from the early phase of CT-PD (sessions 1–10) and every fourth session from the second phase of treatment (11–34). In addition to coding session one for baseline and session 34 for posttreatment, an average of 4.07 (SD = 0.68) of 5 (81%) possible session tapes from phase 1 were coded, and an average of 4.96 (SD = 1.04) of 6 (82.6%) possible sessions from the schema-focused phase were coded. Pattern strength and dispersion scores (described below) take into account the number of sessions available for a given patient. Interrater agreement on all coding categories was good to excellent (ICC = 0.70–0.87). Because agreement was good, the ratings for the two coders on each item of the CHANGE for a given session were averaged. Averaged ratings were used in all analyses.

### CHANGE VARIABLES: EMOTIONAL PROCESSING AND COGNITIVE-AFFECTIVE-BEHAVIORAL-SOMATIC PATTERN VARIABLES

*Emotional Processing* is defined as exploring and questioning issues and emotions related to one’s maladaptive functioning, with some shift in meaning, perspective, and affective response and at higher levels, with an integration of cognitive and affective experiences. Affective arousal without some insight or perspective shift is not considered processing. Rumination, worry, and other perseverative thoughts are also not coded as emotional processing.

Two cognitive-affective-behavioral-somatic pattern variables were created. One assessed patterns related to one’s personality disorder or other maladaptive functioning (labeled *Negative Pattern*), and one assessed more adaptive functioning (labeled *Positive Pattern*). Each pattern included four components or nodes: cognition, emotion, behavior, and somatic functioning. Six CHANGE variables were used to capture the four pattern nodes. Each CHANGE variable is coded for valence (positive, negative) and level (0 = *not present or very low* to 3 = *high*).

A cognitive node variable was created by averaging the scores of three cognitive variables: *View of Self, Hope,* and *Relationships*. This combination captures the cognitive triad: views of self, future, and others (Beck et al., 1979). *View of Self* captures a person’s self-concept and sense of worth. *Hope* captures the person’s expectations for the future and commitment to change. *View of Relationships* is the perceived quality of the person’s interactions with others or one’s view of people in general. The *Emotion* node captures the emotion words expressed in the session, as well as the affective tone and level of arousal. The *Behavior* node describes the number and intensity of adaptive and maladaptive actions the person engaged in since the last session. The *Somatic Functioning* node captures mention of the impact of one’s actions, thinking, or emotions on physiological functioning (e.g., Negative: reporting muscle tension, trouble sleeping, gastrointestinal distress; Positive: reporting feeling calm, relaxed, more peaceful). This category is not coded for physiological responses associated with sickness, surgery, jet lag, and other circumstances not clearly related to one’s psychological functioning.

*Negative and positive pattern strength* scores were computed for the first session and the session closest to session 34 for each patient. These variables yielded measures of overall pattern strength at baseline and posttreatment and provided another indicator of functioning in addition to the SCID-II personality disorder symptom scores. Negative pattern scores were computed by summing the CHANGE scores on the four node ratings [CHANGE ratings for cognition, emotion, behavior, somatic functioning nodes rated 0–3 (0 = *not present to very low*, 3 = *high*)] for a given session. Similarly, positive pattern scores were computed by summing the positive cognitive, emotion, behavior, and somatic functioning nodes. For example, negative pattern strength would be high when a patient describes and elaborates strong views of the self as incompetent, defective, and socially awkward (cognitions, rating = 3), and reports feeling anxiety, loneliness, and sadness (emotions, rating = 3), multiple instances of avoidant coping and social withdrawal (behaviors, rating = 3), and occasional trembling in social situations (somatic, rating = 1). The negative pattern strength in this example is 10.

### MEASUREMENT OF PATTERN ACTIVATION AND DESTABILIZATION: STATE SPACE GRIDS AND GridWare

#### State-space grid

GridWare (Lamey et al., 2004; Hollenstein, 2007) was used to create state space grids for each patient over the course of CT-PD. The two variables used for each axis were: *negative pattern activation* and *positive pattern activation.* These variables were created to operationalize the extent to which the cognitive, affective, behavioral, and somatic nodes of the negative or positive pattern were activated during a given session. These activation scores are different from the baseline and posttreatment pattern scores, which provide a sum of the CHANGE ratings for each of the four nodes (cognitive, affective, behavioral, and somatic), or the overall *pattern strength*. Pattern strength does not provide information on how multimodal the activation is.

The activation scores capture the number of the four nodes activated at a moderate to high level (rating of 2 or 3) on the CHANGE ratings. This sets a clear threshold for defining activation or engagement of each area of functioning (cognitive, affective, behavioral, and somatic). The number of nodes activated (breadth) was of interest because of the therapeutic importance of activating the full network or pattern of pathology to facilitate change (Foa and Kozak, 1986; Teasdale, 1999; Young and Lindemann, 2002; Beck et al., 2004). For instance, multimodal activation and challenges of the beliefs, emotions, behaviors, and somatic responses that maintain a patient’s personality disorder are likely to be more potent than a focus on only one node, such as cognitions. Similarly, developing and exercising multiple nodes of a more adaptive positive pattern can help strengthen and solidify the new learning (e.g., Bouton, 2002; Foa et al., 2006; Craske et al., 2008; Schiepek et al., 2015).

**FIGURE 1:**
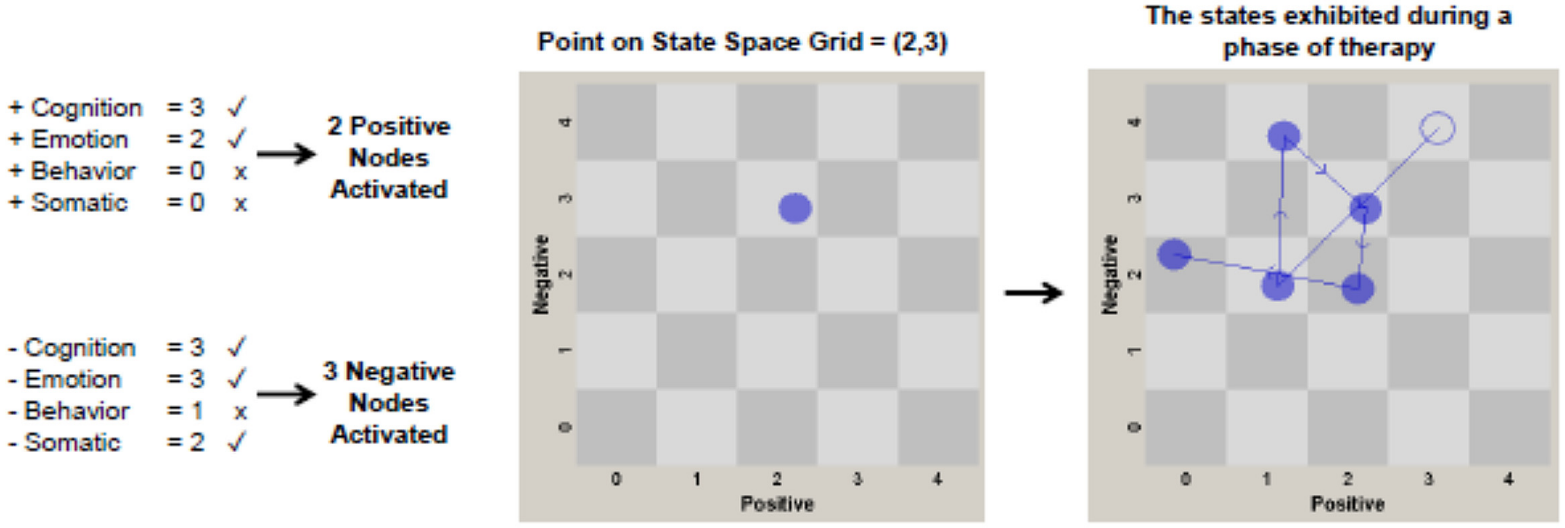
**Chart depicting how CHANGE coding variables (positive and negative cognition, emotion, behavior, and somatic functioning) are converted from raw values for an individual patient to points on the state space grid.** Each CHANGE variable with a check mark (rather than an *x*) was coded at a moderate or high level (score of 2 or 3) and therefore is considered “activated.” The number of CHANGE variables (or nodes of each pattern) activated are then used to plot a point on the state space grid. The number of positive pattern nodes activated corresponds to a value on the *x*-axis, and the number of negative pattern nodes activated corresponds to a point on the *y*-axis. Two treatment phase grids [phase 1 (sessions 1-10), phase 2 (sessions 11-34)] are plotted for each patient. The grids are used to generate dispersion scores for each patient in each phase of treatment. The panel on the right illustrates a patient with a high dispersion score of 0.868 in the schema-focused phase of CT-PD. Low dispersion scores occur when few cells on the grid are visited, as might occur in the earliest sessions of treatment.

To create final positive and negative activation scores, the number of activated positive or negative nodes in each session’s narrative were summed. Each participant had a total score for positive and negative pattern activation per session in a given phase of treatment. These scores could range from 0 (*no nodes activated at a moderate to high level*) to 4 (*all nodes activated above threshold*). GridWare thus assesses pattern *breadth* (number of nodes activated, 0–4), considering also the *strength* of activation (0 = *none to very low*, 1 = *low*, 2 = *moderate*, 3 = *high* on the CHANGE rating scale) of each node in the negative and positive pattern. In the example of high negative pattern strength above, three (cognition, emotion, behavior) of the four nodes were activated at a moderate to high level; thus, the negative activation score would be 3. **Figure 1** illustrates how CHANGE codings are used to create the negative and positive pattern activation scores.

#### Dispersion

Activation scores for each session for each patient were entered into GridWare, with positive pattern activation on the x-axis and negative pattern activation on the y-axis. This provides a visual map of the behavior of the patterns for that person over the course of CT-PD (phase 1, phase 2). To aid analysis, we separated the behavior of the patterns into two phases of CT-PD: symptom reduction (phase 1: sessions 1–10) and schema focus (phase 2: session 11–34). The distribution of the activation scores can be used to calculate the extent of pattern rigidity or variability.

*Dispersion* operationalizes the variance or “spread” of the positive and negative activation scores across the grid for a particular patient in phase 1 or 2 of therapy. More stability is characterized by less movement across the cells of the grid, whereas more variance is characterized by a wider range of movement or distribution across the cells. Dispersion is computed by taking the sum of the squared proportional durations across all cells in the grid, corrected for the total number of cells and inverted so that values range from 0 (*no dispersion* – all behavior in one cell) to 1.00 (*maximum dispersion*- all behavior distributed across different cells). Dispersion is computed by the equation: 1 - [(n∑(di/D)^2^) - 1]/(n - 1). *D* is the total duration (in this case the total number of sessions in that phase), d*i* is the number of sessions spent in a given cell, and *n* is the total number of cells or states in the grid. The right panel of **Figure 1** shows an example of a patient with high dispersion across the sessions in phase 2 of treatment.

## RESULTS

### PRELIMINARY ANALYSES

Neither AVPD nor OCPD diagnostic status predicted posttreatment outcome on the SCID-II or the positive and negative pattern scores, after controlling for respective pretreatment scores. In addition, there were no significant differences between AVPD and OCPD patients on the extent of dispersion or processing in either phase 1 or 2 of CT-PD. Thus, the two Cluster C groups, AVPD and OCPD, were aggregated to increase statistical power.

The first session maladaptive (negative) and positive pattern strength scores were used for baseline and the scores closest to session 34 were used for posttreatment for each patient. Dispersion was calculated for the sessions coded in the early phase of treatment (phase 1: sessions 1–10, excluding baseline) and then in the schema phase (phase 2: sessions 11–34, excluding posttreatment). The highest (peak) level of processing was identified from the first 10 sessions and then from the schema phase of treatment. Because emotional processing often occurs and then decreases, the highest level achieved (peak) more accurately detects shifts in meaning, perspective, and affective responses than mean values, as we have found in previous research (e.g., Hayes et al., 2007a). The modal session number of peak processing was 15 (56% of the sample), and the mean peak processing session number was 18.40 (SD = 4.26). In all cases, the peak processing score preceded the measure of posttreatment outcome.

Descriptive and correlational statistics for all predictor and outcome variables are presented in **Table 1**. Pretreatment personality symptom severity was not associated with any of the predictors, but higher baseline positive pattern scores were associated with more early dispersion. Dispersion in phases 1 and 2 were significantly correlated, as were processing scores in phases 1 and 2. Dispersion in phase 2 was marginally but not significantly correlated with processing in that same phase. It is interesting to note that the negative and positive pattern strength scores were not significantly correlated at baseline or posttreatment, suggesting that the positive pattern strength scores might be more than simply a decrease in the negative scores.

**Table 1:** Summary of intercorrelations, means, and SD for predictor and outcome variables.

Variable	1	2	3	4	5	6	7	8	9	10
1. Dispersion 1		–								
2. Dispersion 2	0.57**	–								
3. Processing 1	0.06	0.05	–							
4. Processing 2	0.31	0.32	0.45*	–						
5. SCID-II pre	-0.18	-0.13	0.04	0.03	–					
6. SCID-II post	-0.39*	-0.53**	-0.14	-0.55**	-0.33	–				
7. Negative pre	0.17	-0.02	0.04	0.08	0.22	-0.41*	–			
8. Negative post	-0.01	-0.30	-0.01	-0.17	-0.14	0.04	0.11	–		
9. Positive pre	0.51**	0.34	0.01	0.23	-0.08	-0.45*	0.18	-0.19	–	
10. Positive post	0.39*	0.62**	0.22	0.64***	-0.09	-0.73***	0.30	-0.29	0.50**	–
Mean	0.62	0.54	1.65	2.12	10.82	4.93	4.68	3.63	1.55	2.28
SD	*0.24*	*0.33*	*0.53*	*0.62*	*2.18*	*3.35*	*1.24*	*1.53*	*1.28*	*2.09*

1 = Phase 1; 2 = Phase 2; SCID-II = Structured Clinical Interview for DSM-IV; Negative = Personality Disorder Pattern; Positive = Positive Pattern; Pre = Baseline; Post = Posttreatment. *p < 0.05, **p < 0.01, ***p < 0.001.

As reported in a previous publication on this sample (Strauss et al., 2006), both personality disorder and depression symptoms decreased significantly with large effects sizes. Paired sample *t*-tests in the current study also revealed that CT-PD was associated with significant pre to posttreatment reductions in maladaptive patterns (negative pattern: mean diff = 1.06, SD = 1.87, *CI* = 0.32, 1.79, *t*(26) = 2.94, *p* < 0.01, Cohen’s *d* = 0.57) and increases in adaptive functioning (positive pattern: mean diff = -0.72, SD = 1.83, *CI* = -1.45-0.0005; *t*(26) = -2.06, *p* < 0.05, *d* = 0.40). Processing scores were higher in phase 2 than in phase 1 (Mean diff = -0.46, SD = 0.65, *CI* = -0.72, -0.20, *t*(26) = -3.64, *p* = 0.001; *d* = 0.71), but dispersion scores did not differ significantly between phases (Mean diff = 0.08, SD = 0.27, *CI* = -0.03, 0.20, *t*(26) = 1.53, *p* = 0.138; *d* = 0.29).

### PREDICTORS OF TREATMENT OUTCOME

Hierarchical multiple regression analyses were conducted to examine dispersion and emotional processing as predictors of posttreatment outcomes. Personality symptoms and negative and positive pattern strength scores at session 34 were examined as outcomes in separate models. In all models, pretreatment scores for a given outcome variable were entered in Step 1. In Step 2, dispersion and processing in phase 1 were entered in the first set of equations, and those same variables in phase 2 were entered in a second set of equations.

Neither dispersion nor processing in the early phase of CT-PD predicted any of the treatment outcomes (see **Table 2**). However, higher scores on dispersion and processing in phase 2 uniquely predicted improvement; together these variables accounted for 49% of the variance in personality symptoms and 64% of the variance in positive pattern strength. Neither dispersion nor processing predicted change in the negative pattern strength (see **Table 3**).

**Table 2:** Hierarchical multiple regression analyses: dispersion and processing in phase 1 predicting therapy outcome variables.

	*B*	SE	β	*t*	*p*	*R*^2^	Δ*R*^2^	Δ*F*	*p* Δ*F*
**SCID-II**
Step 1						0.11	0.11	3.15	0.088
SCID-II pre	0.51	0.29	0.33	1.74	0.088				
Step 2						0.24	0.13	1.93	0.168
SCID-II pre	0.43	0.28	0.28	1.52	0.142				
Dispersion 1	-4.75	2.66	-0.33	-1.79	0.087				
Processing 1	-0.81	1.15	-0.13	-0.70	0.490				
**Negative Pattern**
Step 1						0.01	0.01	0.28	0.599
Negative pre	0.13	0.24	0.11	0.53	0.599				
Step 2						0.01	0.001	0.01	0.987
Negative pre	0.14	0.26	0.11	0.53	0.601				
Dispersion 1	-0.19	1.38	-0.03	-0.41	0.889				
Processing 1	-0.05	0.60	-0.02	-0.08	0.940				
**Positive Pattern**
Step 1						0.25	0.25	8.36**	0.008
Positive pre	0.82	0.28	0.50	2.89**	0.008				
Step 2						0.32	0.07	1.13	0.341
Positive pre	0.68	0.33	0.41	2.07	0.050				
Dispersion 1	1.51	1.79	0.17	0.84	0.408				
Processing 1	0.81	0.68	0.21	1.19	0.246				

SCID-II = Structured Clinical Interview for DSM-IV; 1 = phase 1 (sessions 1–10); Negative = Personality Disorder Pattern; Positive = Positive Pattern; Pre = Baseline; Post = Posttreatment. **p < 0.01.

**Table 3:** Hierarchical multiple regression analyses: dispersion and processing in phase 2 predicting therapy outcome variables.

	*B*	SE	β	*t*	*p*	*R*^2^	Δ*R*^2^	Δ*F*	*p* Δ*F*
**SCID-II**
Step 1						0.07	0.07	1.77	0.196
SCID-II pre	0.42	0.32	0.27	1.33	0.196				
Step 2						0.49	0.42	8.77**	0.002
SCID-II pre	0.37	0.25	0.24	1.50	0.149				
Dispersion 2	-3.59	1.69	-0.35	-2.13*	0.046				
Processing 2	-1.92	0.71	-0.45	-2.72*	0.013				
**Negative Pattern**
Step 1						0.03	0.03	0.62	0.438
Negative pre	0.19	0.24	0.16	0.79	0.438				
Step 2						0.12	0.10	1.16	0.333
Negative pre	0.19	0.24	0.16	0.81	0.429				
Dispersion 2	-1.12	0.99	-0.27	-1.23	0.231				
Processing 2	-0.19	0.42	-0.10	-0.45	0.658				
**Positive Pattern**
Step 1						0.24	0.24	7.05*	0.014
Positive pre	0.78	0.30	0.48	2.66*	0.014				
Step 2						0.65	0.42	12.72***	0.000
Positive pre	0.40	0.22	0.25	1.81	0.085				
Dispersion 2	2.51	0.92	0.39	2.73*	0.013				
Processing 2	1.27	0.38	0.46	3.73**	0.003				

SCID-II = Structured Clinical Interview for DSM-IV; 2 = phase 2 (sessions 11-34); Negative = Personality Disorder Pattern; Positive = Positive Pattern; Pre = Baseline; Post = Posttreatment. *p < 0.05, ** *p* <0.01, ***p < 0.001.

## DISCUSSION

We illustrate how GridWare (Lamey et al., 2004; Hollenstein, 2007), a computer program designed by developmental researchers to study transitions from a dynamic systems perspective, can be used to investigate variables central to psychotherapy research. The state space grid generated by GridWare can depict pattern activity for a given person in a specific phase of treatment or across an entire course of treatment. In our study, the program was used to capture pattern activation and destabilization (measured as dispersion). Patient sessions were coded with the CHANGE coding system (Hayes et al., 2006), which assesses components of pathological and more positive patterns of functioning, as well as emotional processing. Researchers also can choose other combinations of variables relevant to their specific research questions. The stability and variability of the patterns can be depicted and quantified using the measure of dispersion generated by GridWare. These within-individual variation data, which are gathered across a specified time window (e.g., a phase of treatment), can then be used at the group level of analysis to examine questions such as whether and where maladaptive patterns destabilize, what correlates with this increase in variance, and whether variability predicts better treatment outcomes. In this study, the phase 1 and phase 2 pattern dispersion scores for each individual, together with peak processing scores for each phase, were examined in regression equations as predictors of posttreatment outcomes. This illustrates how individual- and group-level data can be combined in simple ways to capture some of the dynamics of therapeutic change and also how coding systems, such as the CHANGE (Hayes et al., 2006) can be used to create therapy process studies from ongoing, or archived clinical trials.

In addition, the findings from this study can contribute to current theories of therapeutic change and fit within a broader framework of general system change. As hypothesized, pattern destabilization and emotional processing during the schema phase of therapy were both significant predictors of improvement in personality disorder symptoms and positive pattern strength at the end of treatment. There was some specificity, as only these variables during the schema phase of CT-PD predicted outcome.

### PATTERN VARIABILITY (DISPERSION)

Avoidant personality disorder and obsessive–compulsive personality disorder are characterized by rigid restriction of affect, avoidance, and perseverative thinking and behaving. As in the treatment of anxiety disorders (Foa et al., 2006), the current study suggests that in the right context and at the right time, activation, and destabilization of the personality disorder-related patterns might be beneficial. Maurer et al. (2011) also illustrated that periods of critical instability can reveal therapeutic transition points in the treatment of AVPD.

Early dispersion was only marginally associated with improvement in posttreatment personality disorder symptoms, whereas dispersion in the schema-focused phase not only predicted more symptom reduction, but also predicted an increase in the strength of the positive, more adaptive pattern. It may be that more variability in a person’s thoughts, behaviors, emotions, and somatic functioning in the beginning of treatment reflects an opening or loosening of the rigid patterns of personality disorders and can set the conditions for further change. Indeed, more destabilization early in treatment was strongly correlated with destabilization in the subsequent schema-focused phase.

It is also possible that more early variability simply reflects less severe personality disorder pathology and rigidity at baseline. However, early dispersion scores were not significantly associated with more symptom severity at baseline, but instead were associated with more positive pattern strength at baseline. Baseline levels of positive resources and those developed over the first 10 sessions of CT-PD (e.g., changes in hope, motivation, self-esteem, and coping) might spark “upward spirals” that can counter or loosen the personality disorder-related patterns (Garland et al., 2010). For instance, in a study of this same trial of CT-PD, Cummings et al. (2012) found that early variability in one’s self-esteem predicted more improvement in personality disorder symptoms and depression. In short, early dispersion might reflect a loosening of the rigid patterns of pathology that can allow for further change.

Dispersion in the subsequent schema-focused phase might capture the turbulence associated with more difficult schema-level change and with the emergence of more positive patterns of functioning. This might be akin to the concept of flickering (Dakos et al., 2013), which involves briefly visiting another attractor or pattern (in this case a new more adaptive pattern of functioning activated and developed in treatment), before the system shifts and stabilizes into the new pattern. Further research is needed to explore whether variability early and later in treatment might capture different facilitative conditions in the therapeutic change process.

### EMOTIONAL PROCESSING

Emotional processing is hypothesized to be a key mechanism of therapeutic change across a range of treatments and clinical disorders (Foa and Kozak, 1986; Greenberg, 2002; Whelton, 2004; Foa et al., 2006; Carey, 2011; McCarthy et al., 2013; Hayes et al., 2014). We further hypothesized that more emotional processing would be apparent at points of destabilization and would predict improvement in personality disorder symptoms, as reported in exposure-based cognitive therapy (Hayes et al., 2007a; Holtforth et al., 2012) and emotion-focused therapy (Pos et al., 2003; Pascual-Leone and Greenberg, 2007) for depression. Indeed, in the current study of patients with AVPD and OCPD (75% of whom had comorbid depression), more pattern destabilization (dispersion) and processing during the schema-focused phase of CT-PD predicted more improvement in personality disorder symptoms and adaptive functioning (positive network). Tschacher et al. (2012) reported similar findings on the importance of emotional activation and clarification, as well as insight, in a group schema-focused therapy for personality disorders.

As proposed in dynamic systems theory (Salvatore and Tschacher, 2012; Schiepek et al., 2015), increased variability and pattern destabilization might index points of transition in psychotherapy that can reveal important predictors of treatment outcome, such as emotional processing (Hayes et al., 2007a; Holtforth et al., 2012). Our findings also contribute to a growing body of literature suggesting that emotional processing, which is thought to play a central role in the treatment of anxiety disorders (Foa et al., 2006), might be also be important in the treatment of other disorders, such as personality disorders (Tschacher et al., 2012; McCarthy et al., 2013) and depression (Pos et al., 2003; Hayes et al., 2007a; Pascual-Leone and Greenberg, 2007; Holtforth et al., 2012)

### POSITIVE GROWTH

Although the schema-focused phase of CT-PD can be destabilizing, both dispersion and processing during this phase predicted improvement in personality disorder symptoms and also in more positive patterns of functioning. This is consistent with research on schema-focused therapy for personality disorders (Young et al., 2003), which suggests that change in schemas and symptomatology mutually reinforce each other and can contribute to the development of new and more adaptive patterns of functioning (Lobbestael et al., 2007; van Vreeswijk et al., 2014).

The strengthening of positive patterns can be underemphasized in traditional psychotherapy for depression, anxiety, and personality disorders relative to the emphasis placed on reducing psychopathology (Dunn, 2012; Carl et al., 2013). However, research in modern learning theory (Bouton, 2002; Foa et al., 2006; Craske et al., 2008) and dynamic systems theory (Thelen and Smith, 1994; Kelso, 1997; van Geert and van Dijk, 2002; Schiepek et al., 2015) suggests that new learning and the development of new attractor states can help solidify change by competing with and preventing a return to old, less adaptive patterns. Our finding of significant change in the strength of the positive pattern at the end of CT-PD is similar to past research showing that cognitive therapy for depression can change the strength and interconnectedness of both negative and positive cognitive self-schemata (Dozois and Dobson, 2001; Dozois et al., 2009). The strength of the new pattern or potential attractor state is likely to be an important predictor of the long-term maintenance of treatment gains.

### LIMITATIONS

A number of limitations should be considered when interpreting the findings from this study. The sample is typical of those reported in clinical trials of long-term treatments for personality disorders, but the sample size is small, and there is a clear need for replication. An important caveat is that some personality disorders, such as borderline personality disorder, are characterized by extreme lability (Zeigler-Hill and Abraham, 2006), and the focus is on stabilization rather than destabilization. Thus, our results might not generalize to treatment for personality disorders other than AVPD and OCPD, which are characterized by particularly rigid patterns of avoidance, worry, rumination, and other types of perseverative processing that inhibit change.

A strength of our approach is that we were able to build a process study into a completed and archived clinical trial, as we have recommended elsewhere (Hayes et al., 2007b). However, we were limited by the design of the original open trial of CT-PD, which did not include a control condition. Therefore, this study is restricted to an examination of within-subject variation, and the findings cannot be attributed specifically to CT-PD. In addition, the assessment of symptoms at baseline, session 17, and session 34 is not ideal for temporal sequencing of pattern destabilization, emotional processing, and change in symptoms and positive functioning. Further, the phase design that we used was imposed on the course of CT-PD based on the focus and content of treatment described in the CT-PD manual (Beck et al., 2004). However, the sessions at which phase 1 and 2 begin and end are approximate. CT-PD focuses on Axis I symptom reduction in roughly the first 10 sessions and then shifts to a focus on schema change. More frequent symptom assessment and more clear phase delineation would have allowed for more precise temporal sequencing of the variables. However, we did attempt to examine negative and positive pattern strength at baseline and session 34 so that they would not be redundant with the measurement of dispersion. We were also careful to identify the phase 2 peak processing levels that occurred before the end of treatment assessment.

The measure of dispersion captures the number of cells visited in the state space grid of GridWare. This variable does not distinguish variability within the personality disorder-related (negative) pattern from movement between that pattern and an alternative more adaptive (positive) one. In other words, the dispersion score could reflect variability within one pattern, across patterns, or within a new pattern. However, the highest dispersion scores are likely to reflect jumps from the personality-related pattern to the positive pattern. GridWare can be used to quantify specific regions of activation and dispersion within and between regions, but that requires more dense sampling of sessions than in the current study.

Another consideration related to session sampling is that because each patient had up to 52 sessions available to code and the CHANGE coding is labor-intensive, not all sessions could be coded. We sampled every other session from the first 10 sessions and then sampled every fourth session thereafter to capture sessions across the most active, schema-focused phase of CT-PD. Although this sampling strategy allows for coverage of the symptom reduction phase (sessions 1–10) and the schema-focused phase (11–34) of CT-PD, it is possible that some important sessions were missed. In addition, the difference in the between-session intervals across the phases might have implications for the extent of dispersion that could be captured by the GridWare program (Lamey et al., 2004). This relatively low density assessment also did not allow for true time series analyses, which could be used to assess the extent of temporal (lag-1) autocorrelation, another important marker of system transition (Dakos et al., 2012a,b; Scheffer et al., 2012). Nonetheless, we did identify two significant predictors of symptom reduction and positive pattern strength: dispersion and processing in the schema-focused phase of treatment.

Our measure of negative and positive patterns is based on coding the content of audiotapes of therapy sessions from a course of CT-PD. The CHANGE coding system (Hayes et al., 2006) assesses the extent to which patients verbalize negative and positive cognitions, emotions, behaviors, and somatic functioning in the session or related to the week before the session. Thus, the coding is limited to patient verbalizations in a given session, and there was no visual information, as would be available with video recordings of the sessions. Somatic functioning could be mentioned in sessions (e.g., feeling nauseous, tense, unable to sleep, or relaxed and calm), but additional physiological measures or reports of physical functioning could have complemented the CHANGE coding of this component.

The pattern scores that we created and the activation and dispersion scores generated by GridWare can be used as individual-level or group-level data to compare phases of treatment, correlate with process variables, and predict treatment outcomes. What we describe is but one approach to the study of change that is relatively straightforward and can be applied to a variety of questions in psychotherapy research. There are also more sophisticated methods for operationalizing networks, patterns, or attractors and connectedness that could be applied to psychotherapy research when sample sizes are large (e.g., Cramer et al., 2010; Borsboom et al., 2011; Schmittmann et al., 2013). There are a range of analytic strategies that can be used to study: (1) variability in time course data (Nesselroade and Ram, 2004; Ebner-Priemer et al., 2009), including time series panel analysis (TSPA), which quantifies session-by-session change in process and outcome variables (Tschacher and Ramseyer, 2009; Tschacher et al., 2012); (2) flexibility and order of patterns of pathology (Schiepek and Strunk, 2010; Fisher et al., 2011; Newman and Fisher, 2013); and (3) network interconnectivity (Dozois and Dobson, 2001; Borsboom and Cramer, 2013), depending on the research questions of interest and the type of data available. A number of tools are also available at the early warning signs of transition toolbox website: www.early-warning-signals.org that can be used to quantify and analyze critical instabilities and critical slowing (Scheffer et al., 2012). In addition, the Synergetic Navigation System (SNS) is an ambulatory and real-time monitoring system that provides intensive assessment of process and outcome variables and tools for time series analyses (Schiepek et al., 2015).

## CONCLUSION

It is important to reiterate that we examined positive and negative patterns, which we conceptualize as attractors, and the role of destabilization in the change process, but using dynamic systems theory as a conceptual framework is not the same as conducting true dynamic systems analyses and modeling. However, the general approach of perturbing an entrenched system and tracking the old and new patterns (or attractors) can provide a useful way of understanding the process of change in cognitive therapy for two entrenched personality disorders, AVPD and OCPD. We illustrated the use of two fairly simple research tools, the CHANGE coding system and GridWare, and how individual and aggregated group-level data can be used to study the process of therapeutic change. We have generated testable hypotheses that can be investigated further in larger samples with more frequent assessments of process and outcome variables, which would allow for more precise temporal sequencing of the variables and a finer degree of resolution.

## Conflict of Interest Statement

The authors declare that the research was conducted in the absence of any commercial or financial relationships that could be construed as a potential conflict of interest.
